# 

**Published:** 1871-08

**Authors:** 


ORIGINAL CONTRIBUTIONS.

Polypus of the Œsophagus. By J. T. Everett. A.M., M.D., Sterling, Ill. ..... 453
An Unique Case of Cancer. By Norman Bridge, M.D., Chicago ................ 456
Cases of Dysentery, Complicated with Malarious Fever.—Strychnia as one of the Remedies, &c. By N. S. Davis, M.D., Chicago. Read to the Chicago Medical Society ........................ 461
The Antseptic Ligature, as applied in the Operation for Varicocele. By Emile Steiger, M.D., Prairie du Chien, Wis... 469

CORRESPONDENCE.

Morphia and Ergot as Parturients. By Geo. H. Vance ........................ 470
Effects of a Large Dose of Veratrum. By T. G Williams, Watertown, Wis. ....... 471
Should Physicians Prescribe Alcoholic Stimulants. By A. Y. McCormick, M.D., Fowler, Ill. .................... 472

CLINICAL REPORTS.

Clinical Notes. By F. K. Bailey, M.D., Knoxville, Tenn. ...................... 474

PROCEEDINGS OF SOCIETIES.

North Iowa Medical Society ............. 486
Minnesota State Medical Society ......... 488
Wisconsin State Medical Society... ...... 490

SELECTIONS.

On the Production of Hæmorrhage, Anæmia, and Emphysema in the Lungs, by Injuries to the Base of the Brain. By C. E. Brown-Sequard, M.D., &c......... 494

BOOK NOTICES.

A Treatise on Diseases of the Nervous System. By William A. Hammond, M.D. ...................................... 497
A Physician's Counsels to Woman in Health and Disease. By Walter C. Taylor. A.M., M.D. ..................... 497
The Modern Operation for Cataract. By Hasket Derby, M.D. ................... 497
The American Journal of Obstetrics and Diseases of Women and Children. Edited by B. F. Dawson, M.D. ........... 497

EDITORIAL.

Preliminary Education ..................... 498
American Medical Association ........... 498
American Medical Association ........... 498
American Association for the Advancement of Science ....................... 502
The Physiological Effects of Severe and Protrac'ed Muscular Exercise; with Special Reference to its Influence upon the Excretion of Nitrogen ............ 502
Mortality for the Month of June, 1871.... 511
Spina Bifida .............................. 513
Money Receipts .................... ...... 513

